# Thyroid dysfunction after hematopoietic stem cell transplantation in children: older age and acute GVHD might be risk factors of thyroid dysfunction during 3 months after HSCT

**DOI:** 10.1186/1687-9856-2013-S1-P142

**Published:** 2013-10-03

**Authors:** Won Kyoung Cho, Kyoung Soon Cho, So Hyun Park, Seung Hoon Hahn, Byung-Kyu Suh

**Affiliations:** 1 Department of Pediatrics, College of Medicine, The Catholic University of Korea, Gyeonggi-do, South Korea

## 

We evaluated short term changes in thyroid function in patients who underwent hematopoietic stem cell transplantation (HSCT) during childhood. And we attempted to find clinical and treatment risk factors for thyroid dysfunction during 3 months after HSCT

We studied 170 patients (102 boys and 68 girls) who underwent HSCT at the Catholic HSCT center from January, 2004 to December, 2009. Patients with a past history of thyroid dysfunction were excluded. The mean age at HSCT were 10.0±4.8. Thyroid function tests were measured before HSCT and at 1, 3 months after HSCT. The normal reference value of T_3_ was 0.8–2.1 ng/mL, free T_4_ was 0.8–2.2 ng/dL, and TSH was 0.17–6.0 mIU/L.

The disease and disease status for HSCT were acute lymphoblastic leukemia (ALL) [first complete remission (CR) = 21, second CR =22], acute myeloid leukemia (AML) (first CR = 74, second CR = 5), chronic myeloid leukemia (first chronic phase = 8), and non-malignant hematological disease (severe aplastic anemia = 32, hemophagocytic lymphohistiocytosis = 3, Fanconi anemia = 5). Out of 170 patients, 169 (99.4 %) underwent allotransplantation. aGVHD (grade II~IV) developed in 79 patients. In 170 subjects, 59 patients received a TBI-based, 81 underwent BU-based, and 30 received reduced intensity conditioning regimens before HSCT. Forty-eight (28.2 %) out of 170 patients showed thyroid dysfunction during 3 months after HSCT (31: euthyroid sick syndrome, 7: subclnical hyperthyroidism, 4: subclinical hypothyroidism, 4: hypothyroxemia, 2: overt hyperthyroidism and 1: high T4 syndrome). In a univariate logistic regression analysis, the Age at HSCT (p=0.003) and acute GVHD (p=0.006) showed relationships with thyroid dysfunction during 3 months after HSCT. We could not find any statistical relationships with sex, diagnosis and conditioninig regimens. In a univariate logistic regression analysis, euthyroid sick syndrome (p=0.006) showed a strong relationships with motality.

In our study, 28.2 % patients experienced thyroid dysfunction during 3 months after HSCT. Euthyroid sick syndrome was most frequent, although subclnical hyperthyroidism, overt hyperthyroidism or subclinical hypothyroidism were also observed. Older age and acute GVHD might be risk factors of thyroid dysfunction during 3 months after HSCT. There was a significant correlation between euthyroid sick syndrome and motality.

